# Whitening Effect of Different Toothpastes on Bovine Dental Enamel: an in situ study

**DOI:** 10.1590/0103-6440202304940

**Published:** 2023-03-06

**Authors:** Anselmo Agostinho Simionato, Rocío Geng Vivanco, Rafaella Tonani-Torrieri, Carolina Noronha Ferraz de Arruda, Fernanda Carvalho Panzeri Pires-de-Souza

**Affiliations:** 1 Department of Dental Materials and Prosthodontics, Ribeirão Preto School of Dentistry, University of Sao Paulo, Ribeirao Preto, SP, Brazil.; 2 Department of Prosthodontics, School of Dentistry, University of the State of Rio de Janeiro, Rio de Janeiro, RJ, Brazil.

**Keywords:** Toothpastes, color, hardness, gloss, roughness

## Abstract

The aim of this *in situ* study was to evaluate color change, surface roughness, gloss, and microhardness in tooth enamel submitted to whitening and remineralizing toothpastes. Fifteen healthy adults (REBEC - RBR-7p87yr) (with unstimulated salivary flow ≥ 1.5 ml for 5 minutes, pH=7) wore two intraoral devices containing four bovine dental fragments (6 x 6 x 2 mm). Participants were randomly assigned and instructed to toothbrush the devices with the tested toothpastes (30 days): CT: conventional; WT: whitening; WTP: whitening with peroxide, and RT: remineralizing toothpaste. A washout period of 7 days was established. Readouts of color, gloss, surface roughness, and microhardness were performed before and after brushing. The results demonstrated no color, gloss, and microhardness differences (p>0.5). The samples brushed with WTP (0.2(0.7) showed higher surface roughness (p=0.0493) than those with WT (-0.5(1.0). The toothpastes did not alter the properties of the dental enamel, except for the roughness. Toothpaste with an abrasive system based on sodium bicarbonate and silica, and that contains sodium carbonate peroxide increased the surface roughness of the enamel.

## Introduction

In recent years, the search for a healthy smile that combines the maintenance of oral hygiene and the esthetic appearance has grown, especially regarding tooth color. Therefore, the use of toothpastes becomes essential not only to prevent biofilm accumulation and polish tooth surfaces but also to remove extrinsic stains caused by pigmentation of the acquired pellicle ^1^.

The demand for increasingly whiter teeth has determined the inclusion of over-the-counter whitening agents ^2^. Among them, whitening toothpastes are the most popular on the market today ^2^. These toothpastes generally promise action by removing extrinsic stains, minimizing tooth color change over time ^1^.

Abrasives and/or chemical agents (such as hydrogen or carbamide peroxides) have been introduced in their composition to promote this effect. However, *in vitro* and *in situ* studies demonstrate that the whitening effect of these toothpastes is not as successful as the whitening protocols performed in-office under a dentist's supervision ^3^ due to the low concentration of peroxides in their composition, their dissolution by the salivary flow, and the short contact time (about 2-3 minutes) with the tooth surface during brushing ^4^. Thus, changes in tooth color may be related to their abrasive effect and not to the peroxide ^5^.

Unfortunately, the use of whitening products can cause morphological changes that compromise the superficial integrity of tooth enamel ^6^ ^,^ ^7^. Its physical and mechanical properties are altered, including changes in surface roughness and microhardness related to tooth wear and mineral loss ^6^. Therefore, randomized clinical studies are necessary to evaluate the possible side effects of these over-the-counter whitening toothpastes.

Tooth appearance also depends on the gloss of the enamel, which is directly related to the morphology of its surface. There is an inverse linear correlation between the surface roughness and the gloss ^8^. Nonetheless, the gloss is not only determined by the microstructural characteristics of the surface. Biofilm accumulation on rough surfaces can also alter the gloss of the enamel ^8^. The bacterial biofilm covers the surface and reduces the gloss ^8^. Therefore, an optimal toothbrushing and the incorporation of appropriate abrasive particles are essential to prevent its accumulation.

Another group of over-the-counter toothpastes are those that provide therapeutic action in relieving dental hypersensitivity ^9^. They present remineralizing agents such as fluoride that can revert or stabilize the enamel mineral loss ^9^ and/or calcium phosphate and arginine that may synergize enamel remineralization ^10^. In addition, the presence of abrasive particles like calcium carbonate, aluminum, calcium phosphate, or silicate can promote tubule occlusion, preventing the movement of intradentinal fluid and thus, reducing the symptoms ^11^.

Fluoride can also inhibit dental demineralization. Fluoride ions replace hydroxyl groups in enamel hydroxyapatite, resulting in the formation of fluorapatite, which is more resistant to acid attack. Changes in the composition of enamel crystals through remineralization can alter the physical and chemical properties of the enamel. The formation of fluoridated apatite results in higher refractive index than the original carbonated apatite, which consequently would alter the light reflection and perception of color ^12^.

Regardless of the promising results of these toothpastes regarding their main mechanism of action, data of crossover clinical trials evaluating their effect on the physical and mechanical properties of tooth enamel are limited to support their indication. The aim of this *in situ* study was to evaluate the color change, surface roughness, gloss, and microhardness in tooth enamel brushed with different over-the-counter toothpastes. The null hypothesis tested was that there would be no changes in the studied properties, irrespective of the toothpaste used.

## Material and methods

### Experimental design

The present in situ study was performed in a block design (volunteers) in which each participant used two removable intraoral devices containing bovine enamel fragments (Each device with four fragments). The sample size was determined using a previous study ^13^, identifying the difference between the means of ΔE for conventional and whitening toothpastes (3.47±3.59 and 2.83±1.30). A total of 14 participants were required (Two-Variance Test, not-equal, 95% of CI, power of 80%).

Eighteen individuals from the community of Ribeirão Preto Dentistry School, University of São Paulo, Brazil, were evaluated for eligibility. The exclusion criteria were the use of illicit drugs, presence of an active carious lesion, periodontal disease, or orthodontic appliance to avoid any interference with the fit of the device or with the salivary flow. One of them had orthodontic braces and two declined to participate, hence they were excluded. Thus, fifteen participants were included in the clinical trial after approval from the Institutional Review Board (CAAE: 79927217.0.0000.5419/ REBEC - RBR-7p87yr). The participants were healthy adults between 20 and 35 years of age (average age 25,93±3,34 years) and presented unstimulated salivary flow ≥ 1.5 ml for 5 minutes with pH = 7 ^14^. A normal unstimulated salivary flow is associated with salivary buffering capacity and with the formation of the acquired enamel pellicle that plays an important role on the prevention of dental demineralization ^15^. In addition, sodium monofluorophosphate requires enzymatic activation by salivary enzymes to release fluoride and sodium fluoride needs to be ionized, thus, a constant and homogeneous salivary flow is necessary ^16^.

The study variables were color alteration, gloss, surface roughness, and microhardness.

### Enamel fragments preparation

One hundred and twenty sound bovine tooth fragments (6 x 6 x 2 mm), without cracks and stains, were cut (Isomet 100 Buehler, Illinois, USA). The fragments were flattened using 320-, 600-and 1200-grit abrasive papers to standardize the thickness (1 mm of enamel and 1 mm of dentin) ^17^. Then, the enamel was polished under water-cooling for 5 min each to standardize the initial surface roughness. The fragments were washed in distilled water using an ultrasonic bath and then sterilized with ethylene oxide at a concentration of 500 mg/L at 50 ºC for 4 hours ^18^.

### Color analysis

For color evaluation, the fragments were placed on a standard white background in a standardized light chamber (Optical Light Cabin Model CL6I-45S, T&M Instruments, São Paulo, Brazil) with a D65 illuminant that simulates the spectrum of daylight. The spectrophotometer (Easyshade, VITA, Bad Säckingen, Germany) was periodically calibrated. After the treatments, new color readouts were performed.

The color change (ΔE_00_) was calculated using the following formula:

$$\Delta E_{00}^{*}=\sqrt{\left(\frac{\Delta L^{'}}{k_{L}S_{L}}\right)+\;\left(\frac{\Delta C^{'}}{k_{c}S_{c}}\right)+\;\left(\frac{\Delta H^{'}}{k_{H}S_{H}}\right)+\;R_{T}\frac{\Delta c^{'}}{k_{c}S_{c}}\frac{\Delta H^{'}}{k_{H}S_{H}}}\;$$

Where ΔL', ΔC', and ΔH' were the differences in lightness, chroma, and hue between two specimens and R_T_ (rotation function) was a function that accounted for the interaction between chroma and hue differences in the blue region. S_L_, S_C_, and S_H_ were the weighing functions for lightness, chroma, and hue components, respectively. k_L_, k_C_, and k_H_ were the parametric factors according to different viewing parameters set to 1.

The variation of WI_D_ (Whiteness Index for Dentistry) was also calculated, as it correlates the data obtained in the CIEDE2000 with color perception ^19^. Positive values indicate lightening and negative values, darkening of the samples. WI_D_ (baseline and after toothbrushing) was calculated with the following formula:

$$WI_{D}=0.511L^{*}-2.324a^{*}-1.100b^{*}$$

The ΔWI_D_ was determined by the difference of final and initial values of WI_D._

### Surface gloss analysis gloss

The gloss analysis (Micro-Gloss 45º, BYK Gardner, Geretsried, Germany) was performed with a readout geometry of 45º to measure the light specularly reflected to the surface ^20^. The values range from 0 to 1000 UB (units of gloss). The gloss was calculated based on the ratio of light reflected by the surface of the fragment and light reflected by the calibration standard at an angle of 45º. For each fragment, five gloss readouts were made before and after brushing. The measurements were expressed as gloss units (GU). The change in gloss (∆GU) was calculated by subtracting the mean initial gloss values from the mean final gloss values (∆GU = GU_f_ - GU_i_) ^21^.

### Surface roughness analysis

The surface roughness (Ra) was measured using a rugosimeter (Surftest SJ-201P, Mitutoyo, Kanagawa, Japan) at a distance of 3.2 mm with 3 cut-offs of 0.8 mm, totaling a readout length of 2.4 mm at a speed of 0.25 mm/s. Three readouts were performed for each fragment: One in the center of the samples and two at a distance of 1 mm to the left and to the right, respectively. The mean of these values was used as the baseline. After the treatments, final readouts were accomplished, as previously stated; and the surface roughness was calculated by the difference between final and initial measurements (ΔRa = Ra_f_ - Ra_i_) ^13^.

### Microhardness analysis

The Knoop microhardness was measured using a microhardness tester (Micro Hardness Tester HMV-2, Shimadzu, Tokyo, Japan) with a pyramid-shaped diamond indenter set to a vertical static load of 25 g for 5 seconds. The largest diagonal of the indention was measured. Three initial readouts were taken for each fragment at defined locations, as described for the surface roughness, and the mean was considered the initial microhardness value. After the treatments, final microhardness readouts were performed. The microhardness alteration was determined by the difference between the final and initial measurements (ΔKHN = KHNf - KHNi) ^17^.

### Intraoral devices preparation and instructions for participants

**Figure 1:**
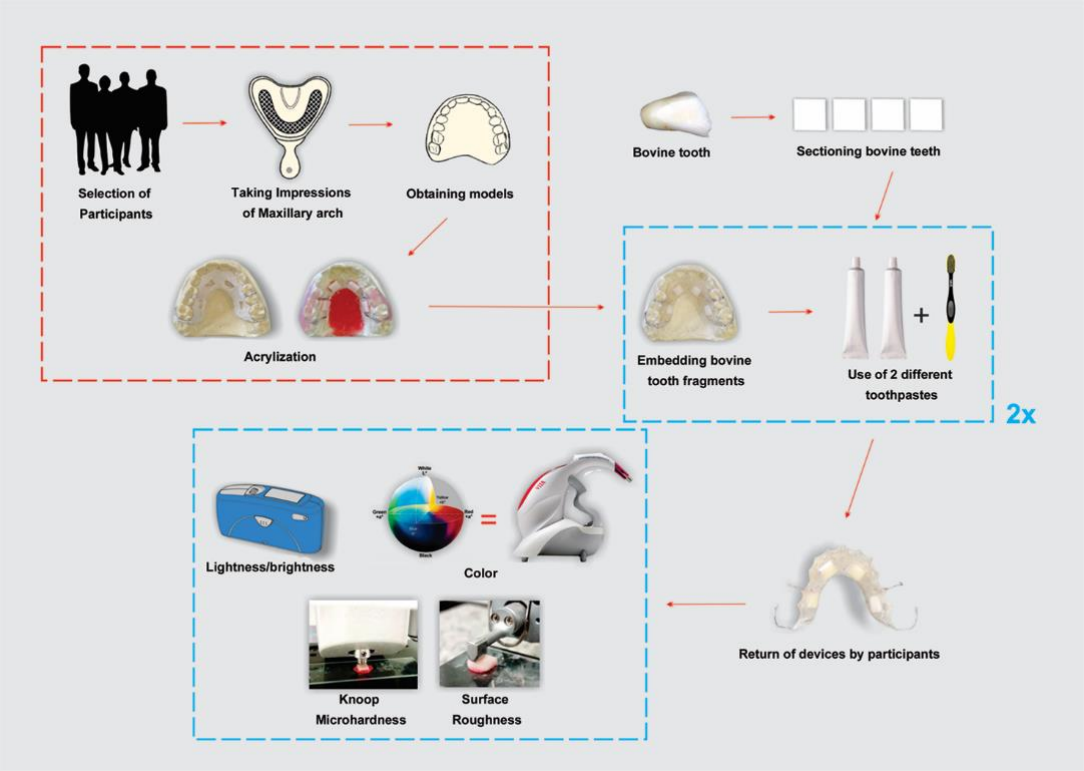
Flow diagram showing how the study was conducted.

For each participant, two oral acrylic resin devices were obtained from the impression (Jeltrate Plus, Dentsply Sirona, York, PA, USA) of the maxillary arch (Figure 1). Each device had four bovine tooth fragments fixed onto the palatine portion: two on the left side and two on the right side of the participant's midline. Box 1 shows detailed information about the toothpastes. CT: conventional toothpaste (Sorriso Dentes Brancos, Colgate-Palmolive, Rio de Janeiro, Brazil); WT: whitening toothpaste (Colgate Luminous White, Colgate-Palmolive, Rio de Janeiro, Brazil); WTP: whitening toothpaste with peroxide (Advance White, Arm & Hammer, Lakewood, CA, USA); RT: remineralizing toothpaste with sodium monofluorophosphate (Regenerate Enamel Science, Unilever, São Paulo, SP, Brazil).

Each toothpaste was packed in 30 g tubes identical in appearance by a researcher, different from the operator so that neither he nor the participant knew which toothpaste was being used, as recommended in a double-blind study. As a randomized and crossover study, all the participants were randomly assigned to brush the enamel fragments with all the toothpastes. The participants used one toothpaste on each side of the device (each side containing two fragments) for 30 days, and a washout period of 7 days was established before and between the treatments ^22^ ^,^ ^23^.

The participants received written instructions explaining the protocol for each phase. They were instructed to brush each side of the device with ten anteroposterior movements for 15 seconds, three times a day ^24^, followed by abundant rinsing with water of the brushed areas and the toothbrush. They were also advised to avoid contamination of the fragments with other toothpaste not tested, brush the devices with the same force as they brush their teeth, and maintain their dietary and oral hygiene habits. The devices were removed during meals to prevent accidents or any bias. In addition, a researcher (A.A.S.) weekly kept in touch with participants to know if they had any problems or difficulties following the proposed protocol to monitor and evaluate protocol adherence.

**Box 1:**
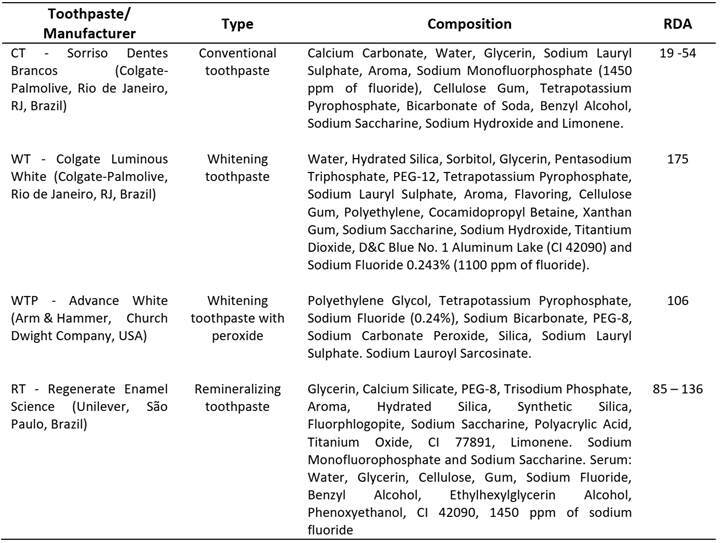
Identification, classification, composition, and abbreviations used for the studied toothpastes.

After the first phase of the study (using a different toothpaste for each side for 30 days with a washout period of 7 days), participants returned to the dental office. They received another device to start a new washout period of 7 days. They also received two new tubes of different toothpastes for the second phase for 30 days. The devices were collected after the second phase, and the fragments were detached. New color, gloss, microhardness, and roughness measurements were performed according to the methodology previously described.

The data were normally distributed for color, surface roughness, gloss, and microhardness (Kolmogorov Smirnov test, p<0.05). Thus, the One-way ANOVA test was performed with the Geisser-Greenhouse correction, with a level of significance of 95%, followed by the post hoc Tukey's test. The WI_D_ values did not show normal distribution; therefore, they were analyzed by Friedmann and Dunn's nonparametric test.

## Results

Eighteen individuals were assessed for eligibility, three of whom were excluded: one because of not meeting the inclusion criteria and two because they declined to participate. Thus, the final sample of this study consisted of 15 participants (4 males and 11 females; average age, 25,93±3,34 years). All of them completed the study.

**Figure 2:**
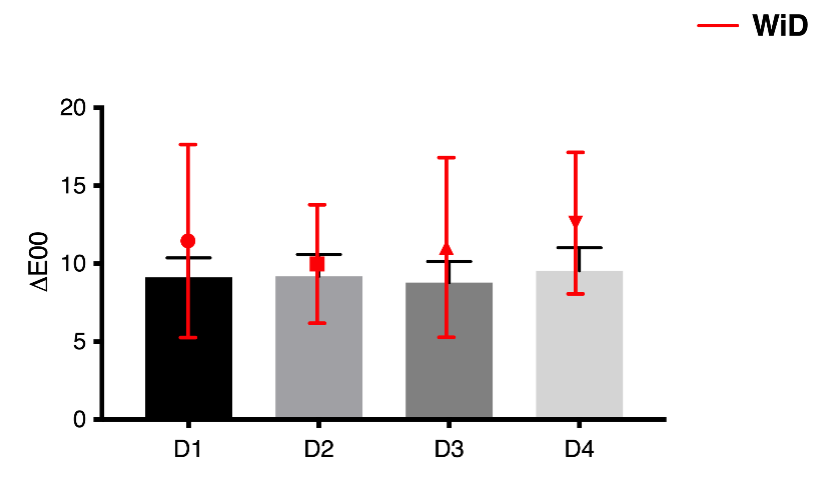
Graph representing superimposition of color change with perceptible whitening of fragments submitted to brushing with the tested toothpastes.

Baseline and final values of the evaluated properties are shown in Table 1. Table 2 shows the comparison of the mean values regarding color alteration (ΔE_00_). There was no difference (p=0.30) among the groups. The whitening index for dentistry (ΔWI_D_) also did not demonstrate a difference (p=0.26) among the groups. However, all the values were positive and higher than ΔE_00_ (Figure 2 and Table 2).

**Table 1:** Baseline and final mean (upper - initial/lower - final) and standard deviation values of L*, a*, b*, gloss (GU), roughness (Ra) and microhardness (KHN) of the enamel fragments brushed with the studied toothpastes.

	CT	WT	WTP	RT
L*	96.44 (2.93)	96.76 (2.8)	96.17 (2.83)	97.28 (2.07)
	98.41 (1.06)	97.49 (2.5)	98.10 (2.0)	98.46 (1.07)
a*	1.21 (0.53)	1.32 (0.58)	1.34 (0.71)	1.37 (0.83)
	3.16 (1.08)	3.95 (2.03)	3.40 (1.07)	3.70 (1.07)
b*	28.78 (2.76)	28.02 (3.30)	29.7 (3.74)	28.51 (4.16)
	19.90 (8.01)	18.13 (7.2)	19.12 (8.02)	17.80 (7.03)
GU	8.09 (1.48)	8.65 (1.68)	8.36 (1.59)	8.35 (1.12)
	9.31 (1.09)	9.88 (2.0)	9.68 (2.13)	9.19 (1.08)
Ra (µm)	1.54 (1.48)	1.61 (1.16)	1.43 (1.01)	1.56 (1.25)
	1.35 (1.2)	1.12 (0.5)	1.64 (1.0)	1.26 (0.8)
KHN	245.1 (40.76)	238.5 (56.64)	259.9 (56.45)	251.4 (45.65)
	423.95 (143.07)	402.54 (103.06)	389.34 (78.02)	407.34 (101.03)

**Table 2:** Mean values (SD) [CI] of ΔE_00,_ ΔWI_D,_ ΔL, Δa, Δb, ∆GU, ΔRa and ΔKHN of the different toothpastes.

	CT	WT	WTP	RT
ΔE_00_	9.12 (1.26)	9.20 (1.39)	8.73 (1.36)	9.54 (1.48)
	[8.7/9.7]	[8.7/9.7]	[8.3/9.3]	[9.0/10.1]
ΔWI_D_	9.56 (5.16)	8.32 (3.16)	9.20 (4.80)	10.50 (3.78)
	[7.6/11.5]	[7.1/9.5]	[7.4/11.0]	[9.1/11.9]
ΔL	1.73 (1.98)	0.58 (1.74)	1.78 (2.11)	1.09 (1.95)
	[0.8/2.4]	[-0.2/1.3]	[0.3/1.2]	[1.1/2.5]
Δa	3.33 (1.47)	3.43 (1.14)	3.35 (1.34)	3.25 (1.09)
	[2.8/3.8]	[2.9/4.0]	[2.8/3.7]	[2.9/3.8]
Δb	-14.92 (1.97)	-14.55 (3.87)	-15.80 (2.63)	-14.72 (3.06)
	[-15.9/-13.9]	[-15.3/-13.8]	[-16.2/-13.3]	[-16.9/-14.7]
∆GU	1.22 (2.38)	1.23 (2.32)	0.83 (2.35)	1.34 (2.10)
	[0.3/2.1]	[0.3/2.1]	[0.5/2.2]	[0.04/1.6]
ΔRa (µm)	-0.11 (1.59)^ab^	-0.49 (1.04)^a^	0.23 (0.72)^b^	-0.17 (1.02) ^ab^
	[-0.7/0.5]	[-0.9/-0.1]	[-0.04/0.5]	[-0.5/0.2]
ΔKHN	193.60 (132.4)	163.96 (92.11)	162.30 (91.46)	143.30 (84.49)
	[144.2/243.1]	[129.6/198.4]	[128.2/196.5]	[111.7/174.8]

Different letters between toothpastes indicate statistically significant differences (p<0.05). Values without letter indication demonstrate no difference (p>0.05) between the toothpastes.

Regarding the color coordinates ΔL and Δa showed positive values after brushing (Table 2), demonstrating increased luminosity (whiter) and reddening of the samples, respectively. While Δb presented negative values, indicating a decrease in the yellow chroma after brushing. Despite this, there was no significant difference (p= 0.31) among the groups.

Table 2 also compares the mean values of ∆GU, ΔRa, and ΔKHN. Regarding ∆GU and ΔKHN, there was no significant difference (p=0.77; p=0.25, respectively) among the groups. Concerning ΔRa, there was a difference (p=0.05) between the toothpaste WT (-0.49+1.04) and the toothpaste WTP (0.23+0.72), which was the only group that showed an increase in the surface roughness of the enamel.

## Discussion

The aim of this *in situ*, double-blind, and crossover study was to compare the effect of different over-the-counter toothpastes on the color, surface roughness, gloss, and microhardness of dental enamel. The null hypothesis was rejected since the toothpastes altered all the properties of the tooth enamel.

Contemporary over-the-counter toothpastes have different formulations and indications for the diverse needs of consumers. Basically, they are composed of abrasives, moisturizers, thickeners, detergents, flavorings, preservatives, and can include therapeutic agents as remineralizing and whitening agents.

The abrasive components in every toothpaste play an essential role in removing extrinsic stains, pigmentations of the tissue, and preventing the accumulation of new stain molecules ^5^. All the toothpastes tested in the present study contain abrasives (CT has calcium carbonate, WT and RT have hydrated silica, and WTP has sodium bicarbonate) that, depending on their abrasiveness (Box 1), can alter the enamel surface. Higher the toothpastes’ relative dentin abrasivity (RDA), higher can be the abrasion and change in the tooth surface that, consequently, may increase the color change. However, despite having different RDAs, there was no difference regarding color alteration between the control group (CT) and the other tested over-the-counter toothpastes.

In addition to the abrasive particles, toothpaste WTP contains sodium carbonate peroxide, which is meant to improve tooth whitening. Nevertheless, WTP did not demonstrate a significant higher color change than the other toothpastes. The fact that peroxide is unstable in aqueous formulations such as toothpastes ^4^ and that it can be quickly diluted by saliva and degraded by salivary enzymes such as peroxidase and catalase, maybe related to the decreasing of its efficacy ^25^. What is more, the over-the-counter whitening toothpastes have a low concentration of peroxide (between 1% and 5%) that remain in contact with the tooth for a short time, so its oxidizing power would be insufficient ^25^.

Although there was no difference between the groups, in all of them it was possible to notice an increase in lightning and reduction in yellow chroma that suggests an increase in whiteness ^26^. Once the organic pigment breaks down, by the peroxide or abrasives components, the molecules found on the dental tissue turn into smaller molecules, reducing the saturation of yellow chroma and presenting a whitening effect ^27^.

The toothpastes also have certain compounds that would affect color perception, which can explain the results of the present study. Toothpastes CT and WTP have tetra potassium pyrophosphate that not only acts as an anti-calculus agent but could also prevent the formation of extrinsic stains and maximize the action of these toothpastes on maintaining the luminosity of the teeth ^28^. The toothpaste RT contains a coloring agent, Blue No. 1 aluminum lake (Color Index 42090), which creates an illusion of tooth whitening. Pigments of optical effect, such as blue covarine, alter the perception of yellowish discoloration ^29^ ^,^ ^30^. They produce a decrease in the yellow chroma and increase the teeth whiteness. Probably, those compounds could have contributed to the results obtained in the b* color coordinate.

The positive change in a* color coordinate may be related to the surface roughness results found in the study. Changes in surface roughness values were negatives for all the tested toothpastes, except for WTP, indicating that the enamel was polished and reduced its surface roughness. Polishing the enamel can reduce the surface roughness but could also increase enamel wear, changing the final color of the tooth ^1^. In the present study, all the toothpastes resulted in positive values of Δa*, meaning a "reddening" of the tooth after brushing. Enamel is a translucent tissue; thus, the tooth color depends on the color of the dentin, which is transmitted through the enamel ^30^. If the enamel is worn, the dentin becomes more exposed due to the reduced thickness of the translucent enamel. The enamel wear by polishing is related to the intrinsic characteristics of the abrasive particles in the tested toothpastes ^1^. Our results differ from those found by Jorge et al., who evaluated the effect of over-the counter agents on the maintenance of color. The treatments produced a significant increase in L* values and decrease in the a* and b* values, meaning that whitening occurred, and probably, there was no enamel wear ^16^.

Regarding WI_D_ index, all the evaluated toothpastes presented positive values of ΔWI_D_, meaning that enamel was perceived as whiter. However, it is important to highlight that the values found in the WI_D_ analysis were higher than those found in the ΔE_00_, which may indicate a greater perception of the color change. This may endorse the fact that the decrease of yellow chroma can lead to a whiter perception of light, and consequently, the teeth color.

Regarding gloss, the results of the present study showed an increase in the gloss for all the toothpastes, without difference among them. Literature shows a relation between gloss to the light reflection on a determined surface, as greater the roughness, lower the surface gloss ^31^. In the present study, surface roughness decreased for almost all the toothpastes. The smoother surfaces increased the gloss values as they can reflect the light in a specular (mirror-like) direction ^31^. Muñoz et al., evaluated the *in vivo* effectiveness of a fluoride dentifrice containing calcium, phosphate and sodium bicarbonate obtaining similar results ^32^. The formation of fluorapatite alters the enamel structure and increases the refractive index, resulting in a glossier appearance ^12^. However, our findings are different from those obtained by Silva et al., who concluded that the whitening toothpastes increased the surface roughness, decreasing the gloss of the enamel ^33^.

Different from gloss analysis, the surface roughness showed difference among the toothpastes. Brushing with toothpaste WTP increased the surface roughness of the enamel, being significant (p=0.049) compared to toothpaste WT. WT has hydrated silica in its composition, and WTP, aside from having silica, has sodium bicarbonate, which is frequently used as a component of abrasive systems ^34^. As the abrasiveness of toothpaste depends on the type, amount, size, hardness, distribution, and structure of the abrasive particles ^1^, the synergy between both abrasive particles may have influenced the results. What is more, WTP contains sodium carbonate peroxide. According to Shamel et al., the difussion of peroxide can cause demineralization creating a rougher surface. Those authors also stated that toothpaste cotanining blue covarine produces less surface abrasion compared to blue covarine-free toothpastes ^35^. Findings in accordance with our results.

Toothpastes containing remineralizing agents can change the microhardness of the tooth enamel surface ^9^ ^,^ ^10^. All the toothpastes used in the present study contain fluoride, and toothpaste RT has soluble phosphate and calcium ^36^. Since toothpaste RT has an additional remineralizing agent, it was expected to have higher microhardness values. Previous *in situ* studies demonstrated that toothpastes containing calcium silicate, sodium phosphate salts and fluoride increase the surface hardness due to the formation of hydroxyapatite on the enamel surface ^37^ ^,^ ^38^. However, caution must be taken when interpreting the results since *in vitro* studies usually use artificial saliva or remineralizing solutions, and different times of application ^37^ ^,^ ^38^. In our *in vivo* study, all the toothpastes increased the microhardness of the enamel, with no significant difference between the groups. The presence of sodium fluoride (NaF) and sodium monofluorophosphate (SMFP) in the toothpastes produced similar microhardness values, and the association of NaF and SMFP would not have advantage over NaF alone as reported by Jhonson ^39^. On the other hand, the calcium silicate and sodium phosphate salts apparently had limited effect. The toothpastes remained in contact with the tooth surface for 15 seconds and according to Hornby et al., those components require a longer exposure time (between 1 and 3 minutes) to produce a more resistant layer of hydroxyapatite ^38^.

The sample size was calculated using a previous study, considering only one of the properties evaluated in the present study (ΔE), as performed by Santana et al. ^40^. Since this is an *in situ* research, it is difficult to have a larger sample size due to the unavailability of some of the participants. However, being a randomized, double-blind, and crossover study minimized bias and ensured the fidelity of the results.

One limitation of the study is the lack of standardization of the applied brushing force by the participants. Different brushing forces affect the abrasive capacity of the toothpastes, enhancing or decreasing their action ^41^. Further, manufacturers inform the composition of the toothpastes, but not the percentage, which is kept confidential, making difficult the discussion of the results. Another limitation of the study was that it was conducted with volunteers living in an area with fluoridated water supply. Carbonate is found in the chemical composition of the teeth, forming carbonated apatite, which is more soluble in acids than hydroxyapatite or fluoridated apatite ^41^. Systemic fluoride and the regular use of fluoridated toothpaste led to the dissolution of carbonated apatite and mineral restructuring of the tooth ^42^, increasing the concentration of fluoridated apatite, thereby decreasing the possibility of seeing differences among the toothpastes.

The toothpastes used in the present study did not alter the properties of the dental enamel, except the roughness. However, the perception of tooth whitening, presented by the whitening index, was higher than the change in color itself. Toothpaste with an abrasive system based on sodium bicarbonate and silica, and that contains sodium carbonate peroxide increased the surface roughness of the enamel.
